# Safe by Design Regulation for Academic Experimentation and Value Conflicts: An Exploration of Solution Directions

**DOI:** 10.3390/ijerph18041554

**Published:** 2021-02-06

**Authors:** Georgy Ishmaev, Pieter E. Vermaas, Dick Hoeneveld, Pieter van Gelder

**Affiliations:** 1 Department of Software Technology, EEMCS, TU Delft, 2628 XE Delft, The Netherlands; 2 Department of Ethics/Philosophy of Technology, TU Delft, 2628 BX Delft, The Netherlands; p.e.vermaas@tudelft.nl; 3 Department of Safety and Security Science, TU Delft, 2628 BX Delft, The Netherlands; d.hoeneveld@tudelft.nl (D.H.); p.h.a.j.m.vangelder@tudelft.nl (P.v.G.)

**Keywords:** Safe by Design, academic experimentation, safety regimes, value conflicts

## Abstract

In this paper, we explore solution directions for the implementation of Safe by Design (SbD) in safety regimes for academic experimentation. SbD is a dynamic and anticipatory strategy for safety regulation in academic research. In this strategy, safety is taken in a broader sense including not only issues of technical precaution of avoiding risks of experimentation but also the societal responsibility of researchers and research institutes of identifying possible future risks. In our research, we have interviewed academic researchers from different disciplines and university support personnel about the factors that enable and limit the possibilities of researchers to implement SbD in safety regimes for experimentation. We articulate our findings in terms of a core set of research values and in terms of conflicts between safety and these research values. And we argue that tools for resolving value conflicts as originating in design for values research can provide directions to solve the value conflicts, and thus help academic researchers to adopt SbD in their experimentation.

## 1. Introduction

Safe by Design (SbD) is a dynamic and anticipatory strategy for safety regulation in academic research taking a prominent place in a variety of research fields and disciplines. The primary focus of SbD is human well-being and environmental protection, thus, it aims to support a clean, healthy and safe environment. Safe by Design thus, not only means a design that allows and conditions safe use for humans across the whole life cycle of the product, from manufacture, construction, transportation and installation, but also safety for the environment through use, maintenance and modification, to decommissioning, demolition and disposal [1]. In this paper, we take Safe by Design as a strategy which aims to achieve the integration of safety in the early stages of academic research and innovation of substances, materials, products and processes in both the precautionary sense of avoiding risk and the anticipatory sense of identifying possible future risks.

SbD is a part of the Dutch governmental environmental policy. With SbD, the government wants to stimulate researchers, designers and companies to take responsibility for risk prevention. While SbD mainly corresponds to the prevention of new risks, research in this area contributes to a better understanding of existing risks as well. Hence Dutch research institutes such as the TU represent aim to implement SbD in their management of research, specifically in the safety regimes for experimentation. Here we define safety regimes as all internal and formal external rules and regulations that are compliant for an academic when performing research.

In this paper, we explore the implementation of Safe by Design in safety regimes for academic experimentation. In academic experimentation, SbD includes the anticipation of risks of new substances in certification and dissemination and risks of new products and services in actual use. In our research, we have interviewed academic researchers from different disciplines and university support personnel at TU Delft about the factors that enable and limit the possibilities of researchers to implement SbD. We articulate our findings in terms of a core set of research values that are endorsed by researchers and other stakeholders in the safety regimes for experimentation. These core values are environmental sustainability, efficiency, commercialisation and researchers’ autonomy. We argue that these core values may come in conflict with the value of safety as envisaged in SbD. For exploring ways to resolve these value conflicts we turn to the literature on design for values [2], focussing on Ibo van de Poel’s [3] work on tools for resolving value conflicts. Practical tools for the resolution of value conflicts in the applied and fundamental research is an emerging area of research. We chose this particular approach from the ethics of engineering, as we consider it to be one of the most developed accounts to date, both in terms of theoretical rigour and successful applications. We argue that these tools can help academic researchers to adopt SbD in their experimentation by providing solution directions to resolving the value conflicts in SbD.

## 2. Methodology

### 2.1. The Interview Phase

For identifying the number and characteristics of safety management regimes for experimentation in research institutes, the research team used literature study, desktop research, review of documents and interviews with stakeholders. We have conducted a number of interviews with researchers and support personnel involved in experimentation, to identify key challenges for the design and adoption of SbD principles in the safety regimes in academia. In our research, we adopted in line with SbD a broad sense of safety, including occupational safety, security, environment, responsible science and the impact of innovation products on society. Therefore, we surveyed all management systems that belong to that broad sense.

As for the field of application, we narrowed that down to Dutch universities, as they are not only the home of much innovative research but also the place where students receive their education and are shaped as future scientists. In that capacity, universities can be considered a model for all research institutes. In the course of the study, we have conducted 19 interviews with the researchers of TU Delft working in the different areas of fundamental research and engineering research including nano-engineering, chemical engineering, civil engineering, electrical engineering, aerospace, and architecture, as well as with the members of the support staff including safety officers (The study was designed to preserve the anonymity of respondents. All participants were informed that no personally identifiable information will be made public, and have signed corresponding informed consent forms for the interviews. The design of the interviews was approved by the Human Research Ethics Committee of TU Delft. Because of the limited number of interviews, more detailed breakdown of the participants’ affiliations and backgrounds could lead to de-anonymization, and therefore is unavailable.).

We have conducted unstructured interviews without a pre-determined set of questions. Our interview methodology was informed by the considerations that the value of safety itself is not a single-dimensional concept, but rather is a relational value, i.e., it is not a mere property of a material (structure, system), but rather a normative judgement whether material (structure, system, experiment setup) is safe for human life or the environment [1,4,5,6].

Another key consideration informing the design of our study is the recurring topic on the distinction between the objective and subjective measurements of safety given that the former is only applicable in the narrow contexts of technical safety [4,5]. However, considering that many safety judgments are made in relation to the objectively perceived values of human life, physical health, or bodily integrity, we treat (expert) subjectivity here as an inherent part of the communication between different contexts of safety in technical and social fields.

Thus, we aimed to identify different values falling under our broad sense of "safety". This suggests the importance of SbD extending beyond the research stage of the innovations process. Such an approach has helped us additionally to identify some of the key values relevant not only to the researchers but also to the affected stakeholders at the later stages of the innovations process–such as environmental sustainability, innovation users’ safety, and environmental safety, (full list of identified values is provided in Appendix A). During the interviews, we have asked the researchers to identify key values (apart from the value of safety) relevant to the implementation of SbD—such as autonomy, research efficiency, creativity, transparency, and uniqueness of innovation. Explication of these concurrent values has made it possible to highlight value conflicts that while not directly related to safety can still hamper implementation of SbD. In the context of our study, we have considered value conflicts as an analytical tool for the identification of key factors facilitating the implementation of SbD in safety regimes at academic institutes.

### 2.2. Plausibility of the Delft University Case

We took the Delft University of Technology as a model for all Dutch research institutes. The assumption is that Delft University safety management regimes and the moral values of the Delft University researchers are representative of the situation in other Dutch research institutes. We expect that this assumption is plausible because most Dutch research institutes have tight cooperation’s and interrelations on operational (e.g., student programs and research facilities) and top management levels. Furthermore, they share many characteristics, amongst them: public funding of almost all institutes, high inter-institute mobility of their researchers [7] and the fact that most universities share the same safety management tool (Lab Servant). Our findings could apply to foreign research institutes, but such was out of the scope of the project.

### 2.3. The Exploration Phase

In our study, we focused on conflicts about the value of safety in safety regimes, with the assumption that resolving them paves the way to introducing SbD into these regimes. Following van de Poel [8] we consider values of varieties of goodness that can be used for moral (or non-moral) evaluation. Given the case where we hold two values “v” and “w” (that do not trump each other), and possible two options “a” and “b”, we may consider this as a case of value conflict iff: (1) value “v” selects the option “a” as best; (2) value “w” select option “b” as best; (3) It is impossible to choose both “a” and “b” (Such typical conflict may occur between, for instance, safety and convenience of an engineering solution, such as in the case of the passenger safety belt. Often, however, value conflict occurs between multiple values and options). On this reading, a value conflict occurs when two or more values provide opposite or contradictory evaluations of the same state of affairs. Thus, if a state of affairs is evaluated as good based on one value, it is in a value conflict by definition bad based on the other value. It needs to be noted, however, that value conflicts deriving from the opposition at the semantic level of values are relatively rare. More often, and especially in the context of engineering and design, value conflicts derive from practical implications of values. Interpreted as such, value conflicts express or correspond to contradictory norms or reason for actions and choices.

In our exploration of ways to resolve the identified value conflicts, we used tools originating in the literature of design for values [3]. We explored the application of these tools on the identified value conflicts, and argue that for a number of value conflicts these tools give useful solution directions.

## 3. General Findings

The interview results have made it possible to identify several key findings in the different research fields, including areas specifically concerned with safety, and areas where safety concerns are an emerging area of attention. It was established that such cross-disciplinary analysis reveals a shared set of values and concerns pertaining to the application of SbD strategies and safety management regimes. This is consistent with the previous findings from empirical studies that viewpoints on safety from different engineering fields can provide a consistent picture, as differences often lie not within the fundamental principles for safety, but in the adaptation to the context [9].

The key areas of attention in the design of SbD approaches are highlighted by the value conflicts, identifiable across the range of different fundamental research and engineering research fields. The general finding is the observation that safety standards and SbD strategies need to be dynamic and ever-evolving to successfully address emerging challenges to human and environmental safety. Another generalisable insight is the need for the development of openness and collaboration between stakeholders separated by the different stages of the innovation process.

In the course of this study, we have interviewed researchers from different fields and members of support staff to identify key values relevant to SbD in the research process. These values were highlighted by the interviewees in the specific context of safety considerations in the research stage of innovation. Furthermore, interviews with the researchers have helped to explicate and map other key values relevant to the broader context of SbD implementations. In this process, we have also been able to identify several major trends defining the dynamics of evolving concerns on safety in the several fields of research: (1) *greening of technologies*; (2) *automation*; (3) *commercialisation*; (4) *administrative burden*.

1. The *greening of technologies* can be broadly characterised as a trend towards the development of more environmentally friendly materials, structures and products. Particular manifestations of this trend are field and context-specific, as different types of innovative solutions may aim at energy saving, fuel efficiency, biodegradability, or other aspects of environmental sustainability. What unifies these developments in different areas of research, be it chemical engineering, civil engineering, or aerospace engineering, is that they push experimentation outside the envelope for which existing safety standards are meant by introducing new types of materials, structures and products that bring with them new types of risks and potential failures.
2. Another significant trend observable in different fields of research is a broad push towards *automation* of computational risk modelling (e.g., structural safety modelling), and implementation of automated safety barriers (e.g., automated recognition of damage in flood defences), enabled by AI and machine learning techniques, defined by the increasing reliance on computational models, automation of processes and tools. This trend, thus, is broadly characterised as an enhancement or even replacement of human performed activities and duties with the software-based solutions puts particular emphasis on the consideration of research efficiency.
3. A third major trend is the *commercialisation* of safety research and safety standard development, as taking place in different fields of innovation. It is not a stand-alone or novel development, given that self-regulation in the emerging technologies and privately funded research are hardly novel developments in themselves. On the one hand, this process is a very welcoming development that can see a wider set of stakeholder engagement in safety research and practices. On the other hand, successful implementation of planning and directing safety (safety strategies) in the context of commercialisation brings up new value conflicts and consequent challenges.
4. The fourth trend confirmed by our interviews is a long-standing trend of an increased *administrative burden* on researchers. This trend has been documented earlier. The US Faculty Burden Survey [10] shows that faculty members who serve as Principal Investigators (PI), actually spend 42% of their federally-funded research time on administrative tasks. Overall, 84% of the PI’s reported that the administrative burdens associated with their research have increased in recent years. Scientific researchers complain about this administrative burden, which includes adherence to safety regimes, and its negative impact on their productivity and ability to do science [11].

These four trends can be captured in terms of conflicts of values held by researchers and safety regimes, and some of these conflicts are relevant to the feasibility of adding SbD strategies to the safety regimes. SbD should not aggravate the value conflicts, for instance, by increase the administrative burden of researchers and thus further limiting their ability to do innovative research productively. Such an aggravation could make researchers and their institutes less willing to accept SbD. Reversely SbD may be welcomed when it alleviates or even resolves value conflicts in current safety regimes. For exploring this feasibility of SbD, we analyse the four trends in terms of values and value conflicts.

## 4. The Four Trends as Value Conflicts

### 4.1. Human Safety and Environmental Safety

The greening of technologies is a major trend emphasising conflicts between the uniqueness of results pushing the edge of safety standards and introducing new types of risks and potential failures. Furthermore, the trend also introduces the potential for conflicts between considerations of human safety and environmental safety at the earlier and later stages of innovation.

Some of the examples of these unexpected risks in nanomaterials are ’carbon black’ nanoparticles used in the production of car tyres. These new nano-materials enable the production of tyres with novel characteristics that enhance fuel efficiency and energy efficiency–desirable properties from the environmental perspective. At the same time in the process of utilisation, there is a potential for the release of "carbon black" nanoparticles in the environment, a factor that may present unknown health and environmental risks.

Other types of concerns are highlighted in aerospace engineering where novel composite materials can help to reduce fuel consumption and emissions of aeroplanes. These novel materials, however, require the development of novel types of tests for structural failures both in the design and maintenance stages, as test techniques used for mainly aluminium structures are not applicable for composite materials.

It is also possible to identify similar value conflicts in civil engineering. For instance, in flood risk management some novel safety challenges can emerge with the development of ’building with nature’ engineering (e.g., mangroves for coastal defences). Such structures require also a reassessment of existing safety standards and anticipation of unknown risks.

### 4.2. Safety and Research Efficiency

The trend of enhancing or even replacing through automation human performed activities such as the modelling of risks by computational means, is driven by the values of research efficiency and uniqueness of innovation, and brings about novel safety concerns as well as emphasises existing ones. (Although it may be noted that automation need not always lead to efficiency. For instance, in aerospace engineering the abundance of empirical safety data allows to test the accuracy of simulations against empirical findings, and can reveal that accurate computer simulations for complex systems may cost more than real-life testing.)

In many fields of research, not just aerospace engineering but also civil engineering, reduction of uncertainties is a crucial element of safety regimes. In this context, software-based computational models provide valuable improvements to the empirical lab and real-world testing. However, in cases where computational risk modelling becomes a replacement for empirical testing, automation does not eliminate uncertainty but rather brings more uncertainty due to the limits of the computational modelling of risks and in contexts where there are no established legal standards for the acceptable use of such models (as in civil engineering). Consistency of safety standards and continuity between different stages of the innovation lifecycle become crucial issues, instrumental for the preservation of the values of safety.

Furthermore, all such computational models are necessarily limited in their scope of prediction, as demonstrated by the collapse of the AFAS football stadium in the city of Alkmaar, the Netherlands, on 10 August 2019. In this case, the investigation revealed design flaws in the roof structure and failure of risk assessment models to account for strong winds [12]. This investigation led to recommendations to systematically use wind tunnel testing for novel structures in addition to predictive models, that is, do not rely solely on digital models.

It is also important to take into account that even successful elimination of human factors by software solutions at the different stages of product (system, structure) lifecycles does not completely eliminate risks due to human factors, but is pushing these risks into the design stage (to the level of code). In the areas of research where robust empirical testing is not available (flood risks, unique structures), AI-assisted modelling helps to derive safety design considerations from simulations (“Safety design” is the concept of applying methods to minimise occupational hazards early in the design process, with an emphasis on optimising employee health and safety throughout the life cycle of materials and processes). However, such simulation alone rarely can provide reliable extrapolations given the difficulty of establishing and identifying causal relations from the statistical models.

This observation should, of course, not be taken as an argument against automation. In many areas of research and innovation, software-based solutions can deliver safety that is unachievable by other types of testing. Automated safety barriers play a crucial role in lab safety solutions, serving as an access control barrier to dangerous materials and equipment for authorised (having necessary training) research personnel [10]. In structural engineering, the automation of safety maintenance also takes a crucial role in the improvement of safety standards. And at the early research and design stages enhanced modelling capabilities provided by AI tools can deliver valuable insights into previously unidentified risks.

### 4.3. Safety and Commercialisation

In the context of academic research commercialisation of safety standards can be regarded as a trend that is driven by the value of efficiency. For instance, commercial suppliers of dangerous materials for research also provide support such as training and certification for lab personnel working with these materials. However, on the larger scale commercialisation of safety standards introduces conflicts with safety by lacking transparency and openness of research.

This conflict between the commercialisation of safety standards and safety is highlighted in the area of civil aerospace engineering, where consistency of integrated safety throughout the whole supply chain is a crucial requirement. In this context, anticipatory safety is regarded not as a property of materials or structures, but as detection of damage before failure. This approach requires industry-wide involvement of stakeholders in the safety research, not only researchers, engineers, and commercial operators of airlines, but also airport operators. Intellectual property and commercial secrets can, however, create gaps in the chains of communication between different suppliers, operators, and aerospace engineering researchers. Given that, at the moment civil aerospace engineering can be considered one of the more advanced fields in terms of safety research (e.g., in aerospace safety design has evolved from ‘fail-safe’ (Here the “safety-principle” is defined as designing in a way so that when a failure does occur, the device will tend to fail predictably to a “safe state.”) principle to “damage tolerance” (Here “damage tolerance” is defined as a property of a product relating to its ability to sustain defects safely until repair can be effected.), in civil structural engineering safety design now evolves towards ‘fail-safe’ principles), these observations provide insights on the potential systemic problems in other fields as well.

This conflict between the commercialisation of safety research and safety also occurs in civil engineering. In the case of novel structures, safety data on stresses and deformations are particularly valuable, which could benefit from shared data repositories. The design of the roof of the Alkmaar football stadium and the design of a parking garage at Eindhoven airport, the Netherlands, which in 2017 also collapsed, can be seen as two cases where safety was compromised by the commercialisation of safety standards.

A somewhat similar trend is observed in the area of research on the health safety of microwaves and radio waves. Here unknown and rare health safety risks can be identified only based on extensive usage data which can be provided only by the commercial companies operating consumer equipment. Current safety standards in this area are based on observable effects such as thermal impact (heating) of biological tissue. However, there is no conclusive evidence for the absence of health effects that cannot be observed in this way. Some (conflicting) interpretations of existing studies point at these additional risks, especially for new consumer products.

### 4.4. Safety and Researchers’ Autonomy

Before considering the value conflict between safety and autonomy due to the administrative tasks of researchers, we want to stress that the importance of administration in research institutes is not disputed by the researchers and lab managers we spoke during our research. There is a shared understanding that administration is intrinsic to research itself, to the validity of findings, to the integrity of its processes, and the justification of research funds. Therefore, it is not the administration itself that is under dispute, but its efficiency and usability for researchers.

Management regimes strive to steer the behaviour of academic researchers and their work processes into the desired direction and to gather information about that direction and those processes. Safety management systems, therefore, have an implicit impact on the research choices and creativity of researchers [10]. For a better understanding of this impact, we focused on safety management regimes; we also made an inventory of other regimes such as Human Resources (HR), Facility Management (FM) and finance regimes, but outcomes were used only as an information source for the safety management regimes. The survey showed that 15 different safety management regimes are present at TU Delft. An additional four "general" regimes were identified and were used as data sources, but they had no active role in safety management as such.

Chances of one researcher needing all 15 safety regimes are almost zero. However, it is not uncommon for a researcher, especially when involved in experimental laboratory work, to deal with 10 different regimes at a time. In that case, up to 17 sets of information have to be entered of which 8 sets may involve double data entries. There is a conflict between safety management regimes and the productivity of researchers, ultimately contributing to constraining the autonomy of those researchers.

## 5. Design for Values and Tools for Resolving Value Conflicts

The implementation of safe by design in safety regimes for academic experimentation has as its aim to broaden the focus of these regimes from issues of technical precaution and avoiding risks in experimentation to possible future risks that may emerge later in the development of technologies. We submit that this goal can be achieved if SbD addresses also the four value conflicts we spelt out in the previous section. Resolving the conflict between safety and commercialisation, for instance, will enable researchers to better explore the future risks of technologies. And addressing the conflict between safety and autonomy may, as said, enlarge the willingness of researchers and their institutes to adopt SbD (Note that some cases of value conflicts fall into a category of conflicting non-commensurable engineering objectives. These cases can be nicely illustrated by the so-called Pareto frontier which is the set of all Pareto efficient allocations. However, as van de Poel [3] argues Pareto principles are not always applicable to the reconciliation of value conflicts in light of two objections. The first is that more value is not always better; sometimes we want to minimise a value (or a criterion for a value), or sometimes we might strive for a specific target rather than for as much as possible. A second objection is that sometimes the desirable degree of attainment of one value may be dependent on the actual attainment of another value).

The literature on design for values [2], specifically Ibo van de Poel’s [3] work on value conflicts in design for values, may provide ways to do address the value conflicts in safety regimes by SbD. For arguing for this possibility, one should take the implementation of SbD itself as a design project, that is, as a design of a new policy for safety regimes that should realise the value of societal responsibility. Van de Poel describes six tools for addressing value conflicts, all in a sense generalisations of tools engineers already use for resolving conflicts between design requirements. We briefly characterise these six tools in this section and then demonstrate in the next section that they can be of use to resolving with SbD the four value conflicts in safety regimes.

### 5.1. Cost-Benefit Analysis

*Cost-benefit analysis* is a rather ubiquitous tool based on economic estimates of the conflicting aspects. In the context of value conflicts this tool requires that the realisation of each of the conflicting values can be evaluated in monetary units, that value commensurability exists between the values, and that their realisation can be compared on a common ratio scale. When these requirements are met, a design that gives the optimal monetary gain realised through the realisation of the conflicting values is the (best) solution to the value conflict.

Cost-benefit analyses may on first sight not be feasible for the design of SbD by all these requirements, and van de Poel discusses in general that this tool is problematic for moral, methodological and practical reasons [3]. Cost-benefit analysis cannot be applied to value conflicts in which one value is taken as imponderable, that is, as a value that cannot be expressed in monetary terms (Another alternative to the cost-benefit analysis is the tool of Multi-Criteria Decision Analysis (MCDA), which can be used to compare not only costs but for instance stakeholders’ preferences. However, like cost-benefit analysis, MCDA also requires commensurability of various criteria which is not always feasible). Autonomy can be such an imponderable value. The tool can also and it cannot be applied to values that are considered as incommensurable. And there are many issues with estimating all the relevant costs to a fair degree. For example, we can try to estimate the environmental damage to wildlife caused by the construction of a dam in terms of the costs involved in mitigating this damage and reintroducing the animals in the affected area. But it could be argued that such an estimate ignores the value of having an intact ecosystem.

### 5.2. Direct Trade-Offs

The tool of direct trade-offs suggests that it might be acceptable to trade off a loss in one value dimension for a gain in another value dimension. The advantage of this tool is that it allows for finding the best or most optimal designs without a need to carry out the problematic and demanding task of expressing values in monetary units. However, the trade-off still tool raises the fundamental issue of unit commensurability of whether a gain in one value dimension can always be compensated by a loss in another dimension [3].

This fundamental issue is certainly problematic if we consider for a product such a central value of as human health and try to compensate a loss in health by an increase in the environmental friendliness of the product. Furthermore, we might encounter what, van de Poel, labels as "taboo trade-offs", which create an irreducible loss because a gain in one value cannot compensate or cancel a loss in the other. However, this does not mean that the trade-off method is not informative in such cases. If moral obligations are interpreted as thresholds for moral values, then below its threshold a moral value cannot be traded off against other values because the moral obligation is more or less absolute. Yet above the threshold, trade-offs may be allowed (It is noteworthy that the Analytical Hierarchy Process (AHP) is an established method for the reconciliation of conflicting objectives. Saaty [13] developed the AHP method to derive weight factors for conflicting objectives, based on the results of pairwise comparisons).

### 5.3. Maximin

The maximin rule tool suggests that in the choice between different options we need to select an alternative that scores best, compared to the other alternatives, on its lowest-scoring value. In the context of engineering design, this method amounts to a kind of "robust design", i.e., to the choice of the design in which the weakest link of that design–the worst scoring value–is relatively strongest, compared to the alternatives [3].

Again, when using this tool, we should avoid making choices for options where the relevant values scores are so low as to make these options morally unacceptable. And, as van de Poel highlights, the use of the maximin tool can also lead to somewhat irrational results especially in the context of designing for safety. For example, in the choice between two solutions where one scores low on safety and low on efficiency, and the second scores almost negligibly lower on safety but much higher on efficiency, the maximin rule tool would irrationally suggest discarding the second option (Apart from maximin rules, minimax rules can be suggested for the choice between different options to minimise the maximum regret).

### 5.4. Satisficing

The tool of satisficing requires that we set for each of the conflicting values a minimum threshold that a design should meet to realise that value sufficiently. When such thresholds can be set, then morally unacceptable design options become those that do not meet the different thresholds. And value conflicts can be dealt with by finding options in which these thresholds are met. The core issue here is that the setting of such thresholds for the values involved is not done arbitrarily but based on relevant moral obligations, codes and standards [3].

### 5.5. Re-Specification

In the design for values approach that van de Poel [14] proposes designers arrive at technologies and products that incorporate the values through an intermediate step of *the specification*. The value designed for is in this specification first translated in specific norms on the technology of the product, or use thereof, that makes that the value is met. Second these norms are translated in (functional or physical) design requirements for the technology or product. This specification is not a logical derivation from the value involved but expresses what the value means in the specific context of the technology or product. If a value conflict exists mainly as a conflict between the specified design requirements, the conflict may be addressed by reconsidering the way the values are specified and arrive at alternative specifications in which the conflict does not occur.

### 5.6. Innovation

Sometimes, however, after all the relevant values are specified and thresholds are established, we may find that the available options do not meet the relevant values, or even that none of the options are morally acceptable. In this case, we need to take further steps beyond moral-philosophical analysis and consider technical means that may enable new, not yet existing options. Engineering innovation can contribute to the resolution of value conflicts if values do not conflict as such, but only in the light of certain technical possibilities [3,8]. Furthermore, the toolkit of design for values can be deployed to consider innovation, not as a general direction, but rather engage in specific types of innovation that ease value conflicts.

## 6. Possible Solution Directions to the Value Conflicts in SbD Implementation

Let us now return to the four value conflicts in current safety regimes for academic experimentation, and give our argument that the design for values tools described in the previous section can help to address these value conflicts when designing SbD implementations for these regimes. First of all, we then need to assume that the values involved in the four conflicts do not necessarily conflict between each other as such, but that conflicts are related to how the values are specified as norms and design requirements. This is certainly the case when we consider the "greening trend" identified during our interviews. Indeed, it would be wrong to think that there is an inherent conflict between the value of human safety and environmental safety as such. Rather these values present a case of what van de Poel [8] characterises as a case of value commensurability and coherence through contingent synergy. In abstract terms, this means that given the state of the world striving for one value may help to achieve another value too.

Second, we have to avoid that a solution direction for addressing one value conflict aggravates one of the other three conflicts. For instance, the conflict between safety and efficiency can be avoided by adding procedures to safety regimes in which it is extensively argued that used models do apply to the products or technologies under analysis. Such a solution would increase the workload for experimenters and thus deepen the conflict between safety and autonomy in experimentation. Extrapolating this argument, it can be observed that the implementation of SbD is better not approached as defining an SbD module that is to be added to safety regimes and defines additional tasks for experimenters: SbD is then typically again deepening the conflict between safety and autonomy. Hence, implementation of SbD is better taken as a redesign of safety regimes.

### 6.1. Addressing the Human and Environmental Safety Conflict

The conflict between human safety and environmental safety is in part a value conflict that plays up in experimentation since assessing the safety risks of innovative green materials and technologies by existing safety standards that do not apply to those materials and technologies, may create environmental risks in the lab. But by the description of this conflict as given in Section 4.1, the value conflict concerns also future human and environmental safety risks in the later stages of the life cycle of the materials and technologies concerned. SbD is actually meant to identify and anticipate these future risks outside the lab, hence if SbD is to be successful it should contain a solution to this first value conflict.

The root of this value conflict between human and environmental safety is the use of existing safety standards to innovative materials and technologies without a clear understanding of whether these standards apply to the innovations. A solution to this is to require a more dynamic approach to safety standards in which it is regularly checked if existing regulation is still capturing the values of human and environmental safety. If the standards are taken as specifications of these values for specific types of materials and technologies, then the tool of *re-specification* seems to be what is needed to resolve the conflict: innovations should be followed by steps in which it is explicitly checked if the existing standards still capture the values of human safety and environmental safety.

We can highlight an illustrative example of "Green Propellants". The initial push of research in this area was driven by the concerns on the safety of lab researchers dealing with highly toxic hydrogen propellants for aerospace. Further research on the alternative non-hydrogen propellants has also brought attention to the high health and environmental risks that the wide use of these chemicals brings. The development of this research program thus also presents an example of innovation that aims to address a wide range of safety concerns pertaining both to human health and environmental safety. This particular example is also illustrative of an evolution of safety concerns from lab safety culture to the scale of broad stakeholder participation.

### 6.2. Addressing the Safety and Efficiency Conflict

The conflict between the values of safety and efficiency occurs when testing for risks can be done by both lab and field experimentation and by computational risk modelling with computer tools, assuming that experimentation is more costly and time-consuming but gives better risk estimates while computational modelling is cheap and fast but introduces new risks. When it is accepted that safety can be monetarised, one has the tools of cost-benefit analysis, trade-offs and maximin available to find solutions to the value conflict. This monetarisation may be an acceptable (and problematic) practice in some domains, say road design, and may thus allow for the replacement for (some) lab and field experimentation by computer modelling of risks. The case of the collapse the roof of AFAS football stadium caused by computational modelling, and the ensuing recommendation [12] to use wind tunnel testing for future designs of such structures, points at an unwillingness in engineering and society to accept such trade-offs: reliance on just computational models for risks assessment is untenable if experimentation leads to more precise risk predictions. This suggests using the tool of satisficing to resolve the conflict between safety and efficiency, where the risks levels that can be obtained by testing designs in lab and field experimentation, count as the maximum threshold.

Satisficing does not exclude that some experimentation can still be replaced by computational modelling of risks. Efficiency can still be realised if that replacement does not introduce new risks. Computational modelling then becomes a tool to make experimentation less elaborate. Say, computer modelling is used to identify the riskier roof designs, such that only a few less risky construction are subjected to wind tunnel testing. Or computational modelling may fully replace experimentation if it is argued that this does not make risk levels becoming higher. For instance, it may be argued that full reliance on computational modelling may introduce new risks but that these risks can be avoided by specific measures. If the extra risks to the roof of that football stadium due to the modelling had been explicitly known upfront, they could have been avoided by specific strengthening or maintenance measures.

### 6.3. Addressing the Safety and Commercialisation Conflict

We propose that the tool of innovation can guide a way out of the value conflict between safety and commercialisation. Commercialisation may incite business models in which firms engaged in safety research derive their profitability from intellectual property and commercial secrets, which block that relevant information about risks are shared with researchers in the lab and with stakeholders in later phases of the life cycle of materials, products and technologies. SbD requires transparency about safety research and found risks throughout these life cycles. Open science may be that innovation, leading firms to which safety research is outsourced to look for alternative business models, say by earning money by the services they provide. Transparency enhanced by open data on safety research, sharing of safety research findings, and collaboration between various stakeholders enables SbD and also enables firms to be co-designing up-to-date and comprehensive safety standards. This suggests that industry stakeholders ultimately can also benefit from sharing of data and research on safety; while enhancing safety standards on an industry-wide scale, it also opens up new markets for commercial firms.

### 6.4. Addressing the Safety and Researchers’ Autonomy Conflict

Finally, the tool of satisficing can help to address the value conflict between the administrative tasks of researchers and their autonomy. Satisficing suggests that the workload caused by the bureaucratic overhead of safety regimes requirements should not exceed a specific limit and that conversely the time and resources experimenters have for doing their research should stay above a limit. Adding just another regime to an already overloaded regime would inevitably result in an even larger value conflict, leading to increased numbers of researchers trying to avoid administrative tasks [15]. In a time of limited resources, efforts to reduce expenses associated with the productivity of the research enterprise should be a top priority for everyone involved in research and research administration. SbD can be implemented successfully within safety regimes for academic research only if the administrative burden of these regimes remain in check.

## 7. Discussion

Based on the findings of the current research we suggest that the implementation of the future safety regime tools should take into consideration value conflicts pertaining to safety in academic research. We suggest that explicit focus on the researchers, incremental prototyping, and close collaboration with the future users and management in the design of such tools, are critical design elements that can take into account values and value conflicts relevant to the successful SbD strategies. The autonomy of the researchers, freedom of innovation and openness of the safety design process, are values that can be facilitated in the process of such collaborative design. Close involvement of the researchers in the iterative and inclusive design process not only ensures the relevance of such tools for the future users facilitating smooth adoption but also provides avenues for the early identification of emerging safety concerns. We, therefore, see a fruitful collaboration between safety research and research on design for values, for finding those solution directions to value conflicts and for enabling the development of these directions in actual implementations in and beyond academic experimentation. Given the dynamic and ever-evolving nature of the safety concerns, the inclusion of the wider set of stakeholders can enable facilitation of the holistic SbD strategies. Focus on value conflicts resolution can promote successful implementation of safety strategies further down the innovation lifecycle, facilitating collaboration between researchers, industry stakeholders, and regulators.

## 8. Conclusions

In this paper, we explored solution directions for the implementation of SbD in safety regimes for academic experimentation. We have reported our findings from interviews that we conducted with academic researchers from different disciplines and university support personnel about the factors that enable and limit possibilities of researchers to implement SbD in safety regimes for experimentation. These finding could be summarised in terms of four values that in current safety regimes conflict with the value of safety, namely environmental safety, efficiency, commercialisation and autonomy of researchers. Successful implementation of SbD in safety regimes should address these conflicts to become acceptable to researchers and their institutes. Finally, we introduced six tools for resolving value conflicts that originate in research on design for values and argued that these tools can help SbD with finding directions to solve the value conflicts. Hence, design for values may support the introduction of SbD in academic experimentation.

The tools that research on design for values is creating may also more generally be of support to the safety of technologies, products and processes. In this paper, we focused on conflicts between safety and four other values in safety regimes for academic experimentation. Yet beyond these regimes, other conflicts exist as well, as when implementations of safety measures hamper the usability of products and technologies, or when transparency for supporting safety undermines the need for security, as may occur in the chemical industry (e.g., [16]). The design for values approach and its tools for addressing value conflicts may also here be of help in the search for solution directions.

## Appendix A

List of key values pertaining to the implementation of SbD, as highlighted by the interviewed researchers working in the areas of nanoscience, nano-engineering, chemical engineering, civil engineering, electrical engineering, aerospace, and architecture:

- human safety (all respondents);
- environmental safety (all respondents);
- efficiency (all respondents);
- scalability (all respondents);
- transparency, regarding the openness of safety research (all respondents);
- innovation uniqueness (chemical engineering, nanoscience, civil engineering);
- aesthetics (structural engineering, architecture);
- researchers’ autonomy, independence (fundamental research);
- creativity freedom (fundamental research, civil engineering, architecture).
